# Differential Roles of ERα and ERβ in Normal and Neoplastic Development in the Mouse Mammary Gland

**DOI:** 10.1371/journal.pone.0113175

**Published:** 2014-11-18

**Authors:** Rajendra G. Mehta, Michael Hawthorne, Rajeshwari R. Mehta, Karen E. O. Torres, Xinjian Peng, David L. McCormick, Levy Kopelovich

**Affiliations:** 1 IIT Research Institute, Chicago, Illinois, United States of America; 2 GenUs Biosystems, Northbrook, Illinois, United States of America; 3 Division of Cancer Prevention, National Cancer Institute, Rockville, Maryland, United States of America; Baylor College of Medicine, United States of America

## Abstract

The present experiments were performed to determine the roles of estrogen receptors α and β (ERα and ERβ) in normal and neoplastic development in the mouse mammary gland. In wild-type mice, *in* *vivo* administration of estradiol (E) + progesterone (P) stimulated mammary ductal growth and alveolar differentiation. Mammary glands from mice in which the ERβ gene has been deleted (βERKO mice) demonstrated normal ductal growth and differentiation in response to E + P. By contrast, mammary glands from mice in which the ERα gene has been deleted (αERKO mice) demonstrated only rudimentary ductal structures that did not differentiate in response to E + P. EGF demonstrates estrogen-like activity in the mammary glands of αERKO mice: treatment of αERKO mice with EGF + P (without E) supported normal mammary gland development, induced expression of progesterone receptor (PR), and increased levels of G-protein-coupled receptor (GPR30) protein. Mammary gland development in βERKO mice treated with EGF + P was comparable to that of wild-type mice receiving EGF + P; EGF had no statistically significant effects on the induction of PR or expression of GPR30 in mammary glands harvested from either wild-type mice or βERKO mice. *In* *vitro* exposure of mammary glands to 7,12-dimethylbenz[a]anthracene (DMBA) induced preneoplastic mammary alveolar lesions (MAL) in glands from wild-type mice and βERKO mice, but failed to induce MAL in mammary glands from αERKO mice. Microarray analysis of DMBA-treated mammary glands identified 28 functional pathways whose expression was significantly different in αERKO mice versus both βERKO and wild-type mice; key functions that were differentially expressed in αERKO mice included cell division, cell proliferation, and apoptosis. The data demonstrate distinct roles for ERα and ERβ in normal and neoplastic development in the mouse mammary gland, and suggest that EGF can mimic the ERα-mediated effects of E in this organ.

## Introduction

Estrogen action in target tissues is mediated by binding to specific nuclear receptors, estrogen receptors α and β (ERα and ERβ) [1]. ERα and ERβ are ligand-regulated transcription factors, and each has several isoforms; these receptors play key roles in the regulation of cell proliferation and differentiation in the breast, uterus, and other tissues [2]. Following estrogen binding, ERs bind to estrogen response elements (EREs) in the promoter regions of estrogen-responsive genes [3], resulting in a cascade of downstream responses.

Although murine ERα and ERβ demonstrate 97% sequence homology at the DNA binding domain [4], the two receptors are clearly distinct. The genes for ERα and ERβ are located on different chromosomes [5]; the two receptors demonstrate different distribution patterns in estrogen target tissues; and the receptors appear to regulate transcription via different mechanisms. ERα and ERβ also have different ligand binding affinities and respond independently to ligand binding [6]. Most importantly, activation of ERα results in substantially different effects in estrogen target tissues than does activation of ERβ).

The contrasting effects of ERα activation and ERβ activation appear to be both organ-specific and context-dependent [6]. In the normal breast, ERβ is more abundant than is ERα; recent data suggest that high levels of ERβ can down-regulate the expression of ERα [7]. Activation of ERα supports cell proliferation in the breast and other tissues [8]; ERα content has been proposed as a marker to distinguish hormone-dependent breast cancers from hormone-independent breast cancers [5]. By contrast, activation of ERβ is antiproliferative in the breast and other tissues [9]. Because ERα and ERβ demonstrate both qualitative and quantitative differences in activity, understanding their distinct roles in normal and neoplastic mammary growth and development may provide insight into estrogen action in breast cancer, and may also identify useful molecular targets for breast cancer prevention and therapy.

Previous studies have demonstrated cross-talk between the ER and EGFR pathways in both normal and neoplastic development in the mammary gland [10]. Implantation of EGF pellets supports normal mammary gland differentiation in ovariectomized mice, as demonstrated by the development of terminal end buds [11]. In addition, EGF upregulates the expression of G-protein-coupled receptor 30. (GPR30) in two human breast cancer cell lines (SK-BR-3 and BT20) that are negative for ERα; GPR30 has been proposed to play a key role in the development of tamoxifen resistance in breast cancer cells [12]. In the uterus, estrogen upregulates EGFR, and EGF mimics the growth stimulatory activity of E [13], [14]. When considered as a whole, these data suggest that EGF mimicry of estrogen-mediated function(s) may result from (or be associated with) EGF up-regulation of GPR30 [15].

Although comparative studies of the roles of ERα and ERβ in breast cancer have most commonly been performed using ER+ and ER– cell lines as experimental models, αERKO and βERKO mice have been used to understand differential functions of ERα and ERβ [16]. Previous studies have indicated that in the absence of ERα mammary glands do not develop, only rudimentary primary duct is observed in mice, whereas mammary glands from young 3–4 week old female βERKO mice were indistinguishable from the control wild-type mice [17], [18]. However in βERKO mice where ERα is intact there is no adverse effect on the development of mammary glands [17]. Mammary carcinogenesis studies in ERKO mice have not been previously reported. Studies in mammary glands harvested from ERα knockout mice and ERβ knockout mice may provide additional insights into the distinct roles of ERα and ERβ in the development of normal and neoplastic mammary gland [4].

## Materials and Methods

### Estrogen Receptor Knockout (ERKO) Mice

Colonies of αERKO and βERKO mice were developed and maintained in our laboratories using breeding pairs provided through the courtesy of Dr. Dennis Lubahn (University of Missouri, Columbia, MO). Both ERKO models were originally developed on a C57BL6/129SV background; C57/BL6 mice were used in the present studies as wild-type (WT) controls. All mice were genotyped prior to use in order to confirm their ER status. This study was carried out in accordance with the recommendations in the Guide for the Care and Use of laboratory animals of the National Institute of health. The experimental design and protocol were approved by the IIT Research Institute’s Institutional Animal Care and Use Committee (IACUC).

### Mammary Gland Organ Culture (MMOC)

Procedures used to evaluate the effects of hormones, growth factors, and other agents on the *in* *vitro* differentiation of the mouse mammary gland and on the induction of preneoplastic lesions have been described in detail [19], [20]. Briefly, 4-week-old female mice (C57/BL, αERKO, or βERKO) that had been pretreated with 1 µg estrogen and 1 mg progesterone for 9 days were sacrificed by CO2 asphyxiation and thoracic pairs of mammary glands were excised aseptically. Mammary glands were then incubated on silk rafts in serum-free organ culture using chemically defined Waymouth medium.

### Effects of *in* *vivo* preconditioning with EGF on mammary gland differentiation in ERKO mice

In WT mice, *in* *vivo* pre-treatment with E (1 µg) and P (1 mg) is required to support mammary gland responsiveness to hormones in organ culture [16]. Because mammary ducts from αERKO mice are rudimentary and do not respond to hormones, we evaluated the effects of EGF (as a substitute for estrogen) on the *in* *vivo* expansion of mammary ducts in the empty fat pad. WT and ERKO mice were treated with 25 ng EGF+1 mg P daily for 5 consecutive days; mammary glands were collected and processed as whole mounts for evaluations of morphology (structural expansion of mammary ducts and formation of end buds). The effects of EGF + P on mammary gland morphology were compared to the effects of E + P.

### Effects of Hormones on Induction of Preneoplastic Mammary Lesions (Mammary Alveolar Lesions; MAL) by a Chemical Carcinogen

To study the effects of hormones on MAL development, freshly harvested mammary glands were incubated in Waymouth’s MB 752/1 medium supplemented with insulin (I; 5 µg/ml), prolactin (PRL; 5 µg/ml), aldosterone (A; 1 µg/ml), and hydrocortisone (F; 1 µg/ml) for 10 days (defined as the growth promotion phase). On Day 3 of the growth promotion phase, glands were exposed to the polycyclic aromatic hydrocarbon carcinogen, 7,12-dimethylbenz[a]anthracene (DMBA; 2 µg/ml) for 24 h. After completion of the 10-day growth promotion phase, glands were grown for an additional 14 days in medium containing insulin only (5 µg/ml; regression phase); the regression phase permits microscopic visualization of preneoplastic mammary alveolar lesions (MAL). At the end of the 24 day culture period, glands were fixed in formalin, stained with alum carmine, cleared in xylene, and evaluated microscopically [16]. Glands were scored for the incidence and multiplicity of MAL.

### Immunohistochemistry

Formalin-fixed, paraffin-embedded tissue sections (4 µm) were deparaffinized and rehydrated for immunohistochemistry. Antigen retrieval was performed by microwaving tissue sections in 10 mM citrate buffer for 6 min. After rinsing with PBS, nonspecific staining was blocked by incubating slides in blocking reagent (Vector Laboratories, Burlingame, CA). Tissue sections were incubated with primary GPR30 antibodies (Santa Cruz Biotechnology, Santa Cruz, CA), rinsed with PBS, and incubated with the appropriate biotinylated goat anti-rabbit/anti-mouse antibody (Vectashield Elite, Vector). Immunoreactivity was visualized using diaminobenzidine (Sigma-Aldrich, St Louis, MO) as a chromogen. Sections were counterstained with hematoxylin, and were examined microscopically.

### Microarray analyses

Mammary glands were collected from five mice per group (WT, αERKO, βERKO) and snap-frozen in liquid N2 for microarray evaluations using the Codelink Mouse Whole Genome Array (Applied Microarrays, Tempe, AZ). Mammary glands containing DMBA-induced MAL were harvested from WT and βERKO mice; because glands from αERKO mice were MAL-free, no samples could be harvested for molecular analysis.

Purified total RNA was extracted individually from each gland using the Ambion RiboPure RNA isolation kit (Life Technologies, Carlsbad, CA) and was quantitated using the OD260/280 ratio for each sample. Equal mass amounts of total RNA from each gland were pooled to yield a sample representing RNA from mammary glands from every mouse in each group. Arrays were hybridized at 37°C for 18 h in a shaking incubator, washed, and stained with Cy5-Streptavidin dye conjugate (Amersham Biosciences, Piscataway, NJ) for 30 min. Rinsed and dried arrays were scanned with a G2565.

Microarray Scanner (Agilent Technologies, Santa Clara, CA) at 5 µm resolution. Scanned images from arrays were processed using CodeLink Expression Analysis software (Applied Microarrays, Tempe AZ); data was analyzed using GeneSpring GX v7.3 software (Agilent Technologies, Santa Clara, CA). To compare individual expression values across arrays, raw intensity data from each probe were normalized to the median intensity of the array. Genes with values greater than background intensity in at least one group were evaluated using Principal Component Analysis (PCA) and hierarchical clustering to identify differences in global gene expression between groups. Genes were further filtered for >2 fold differential expression in each comparison (αERKO/WT, βERKO/WT, and αERKO/βERKO). Venn diagram analysis was used to identify overlaps of differentially expressed genes, and to identify MAL-specific genes that were responsive to ERα or ERβ. Signaling pathways enriched with differentially expressed genes were identified as those with overlap p values of <0.001 on a hypergeometric distribution.

Microarray data were also analyzed using Ingenuity Pathway Analysis software (Ingenuity Systems, Redwood City, CA). Comparative gene expression ratios were calculated for αERKO/WT, βERKO/WT, and αERKO/βERKO in order to identify ERα- and ERβ-responsive genes and enriched canonical pathways. Nodal networks containing ER or including cellular growth and proliferation functions were separated for each ERα-responsive and ERβ-responsive gene.

### qRT-PCR

Quantitative RT-PCR was performed as previously described [21], using the MyiQ real-time PCR detection system (Bio-Rad, Hercules, CA) and iQ SYBR Green PCR Supermix (Bio-Rad, Hercules, CA); assays were performed using manufacturer’s instructions. Progesterone receptor (PR) expression was analyzed by normalizing PR expression to the quantity of total RNA used for RT-PCR analysis. Primers used for PCR analysis of PR were 5′-ATGAAGCATCTGGCTGTCACTA-3′ (forward) and 5′-AAATAGTTATGCTGCCCTTCCA-3′ (reverse).

## Results

### Mammary gland development in response to estrogen + progresterone requires ERα but not ERβ

**Figure 1:**
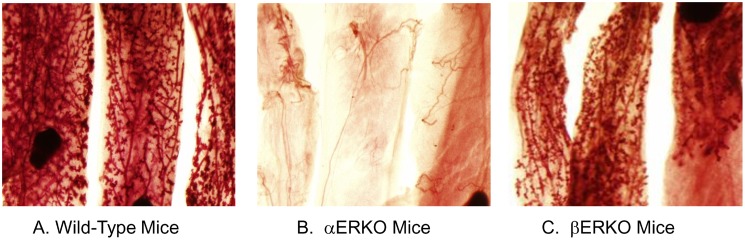
Effects of pretreatment with estradiol and progesterone on the mammary gland development in MMOC. WT, αERKO and βERKO mice were treated with 1 µg estradiol and 1 mg Progesterone subcutaneously for 9 days. The thoracic pairs of glands were dissected and cultured in serum free Weymouth’s medium for 10 days in the presence of insulin (1 µg/ml), prolactin (5 µg/ml), aldosterone (1 µg/ml) and hydrocortisone (1 µg/ml). Results show that αERKO mice pretreated with E and P do not respond to hormones, no ductal expansion or alveolar development is observed (panel B), whereas WT and βERKO mice respond to E plus P treatment and to hormones in MMOC.

Previous studies have demonstrated that mammary gland development in untreated αERKO mice is rudimentary in comparison to mammary gland development in either WT mice or untreated βERKO mice [11]. To determine the effects of mammotropic hormones on mammary gland development in these mice, WT mice, αERKO mice, and βERKO mice were pretreated with E (1 µg) plus P (1 mg) for 9 days, and then cultured *in* *vitro* in the presence of growth promoting hormones. *In* *vivo* administration of E + P followed by the 10 days of *in* *vitro* incubation in organ culture induced mammary ductal expansion and formation of alveoli in both WT C57/BL6 mice (Figure 1, panel A) and βERKO mice (Figure 1, panel C). By contrast, pretreatment with E + P followed by *in* *vitro* incubation with growth promoting hormones failed to induce ductal expansion or end bud development in the mammary glands of αERKO mice (Figure 1, panel B). These results demonstrate that ERα is required to support the induction of normal mammary gland growth and development by E + P; by contrast, induction of mammary ductal expansion and end bud development land in response to pretreatment with E + P can occur in the absence of ERβ.

### EGF can replace E in supporting normal mammary gland development

**Figure 2:**
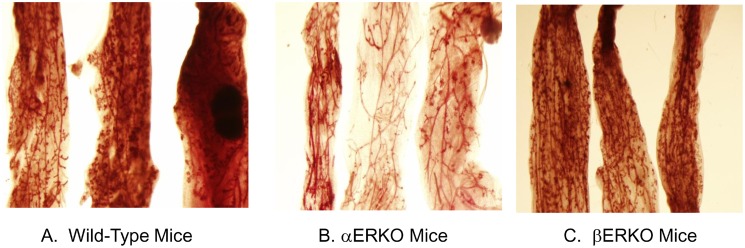
Effects of pretreatment with EGF and progesterone on the mammary gland development in MMOC. WT, αERKO and βERKO mice were treated with 25 ng EGF and 1 mg Progesterone for 5 days s.c. The glands were dissected and cultured in serum free Weymouth’s medium for 10 days in the presence of insulin (1 µg/ml), prolactin (5 µg/ml), aldosterone (1 µg/ml) and hydrocortisone (1 µg/ml). Results show that mice pretreated with EGF and P can replace pretreatment with Estradiol and progesterone for ERKO mice. A. WT, B. αERKO, C. βERKO Mice.

In consideration of the reported crosstalk between ER and EGFR in mammary epithelial cells [13] we hypothesized that EGF + P could replace E + P in stimulating mammary gland development *in* *vivo*. This hypothesis was addressed by comparing the effects of EGF + P on ductal expansion in mammary glands from WT mice, βERKO mice, and αERKO mice. As shown in Figure 2, WT mice (panel A) and βERKO mice (Panel C) responded to *in* *vivo* treatment with 25 ng EGF+1 mg P in a manner that was similar to their responses to E + P (Figure 1). Although the effects of EGF + P in αERKO mice were less prominent than were seen in either WT or βERKO mice, ductal expansion throughout the mammary fat pat was also seen in αERKO mice treated with EGF + P (Figure 2, panel B). This finding is in contrast to the lack of ductal expansion αERKO mice receiving *in* *vivo* treatment with E + P (Figure 1, panel B). These data suggest that EGF + P can substitute for E + P in inducing normal mammary gland growth and differentiation in both WT mice and in mice lacking ERα or ERβ.

### EGF induces expression of progesterone receptor and GPR30 in αERKO mice

**Figure 3:**
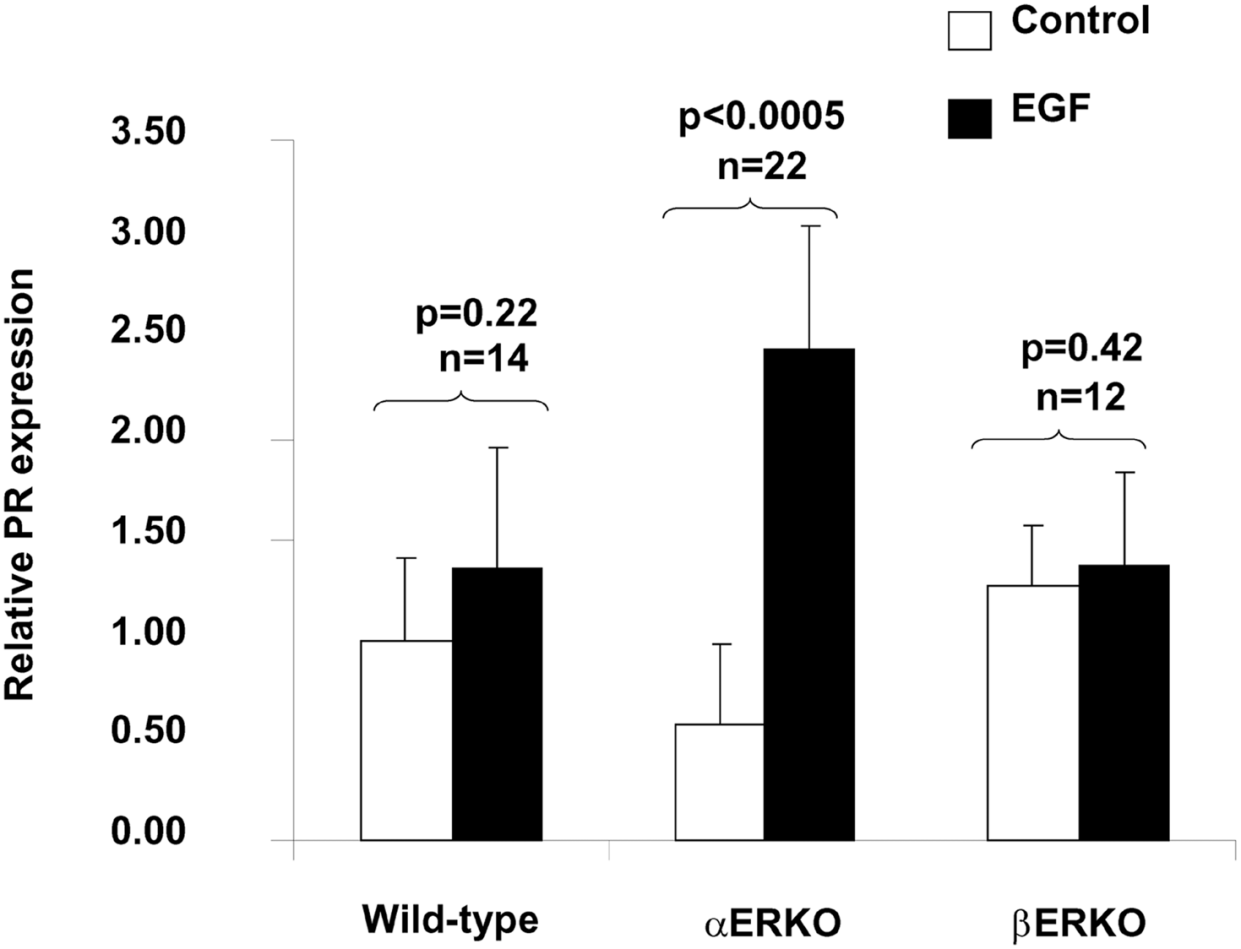
Effect of EGF treatment of mice on the progesterone receptor (PR) mRNA expression in the mammary glands. Mammary glands were isolated from WT, αERKO and βERKO mice pretreated with or without EGF (25 ng/mouse/day, 5 days) and homogenized in Trizol (Invitrogen). Total RNA was isolated and subjected to quantitative RT-PCR analysis. Primers were designed to recognize both PR-A and PR-B. PR mRNA levels in the control samples were set as 1 after normalized to the amount of total RNA. The results are expressed as a mean ± SEM, αERKO**p<0.0005 (n = 22) in comparison to control, whereas the induction of PR mRNA in WT and βERKO mice was not statistically significant between EGF treated and untreated glands (one-way ANOVA).

PR is an estradiol-inducible target gene [22]. To determine whether EGF can replace E in inducing *in* *vivo* expression of PR in the mammary gland, groups of WT, αERKO, and βERKO mice received daily subcutaneous injections of EGF (25 ng/dose) for five days. As seen in Figure 3, EGF induced a ∼4.5-fold increase in mammary PR expression in αERKO mice (p<0.01), but had no statistically significant effect on PR expression in mammary glands of either WT mice or βERKO mice. These data demonstrate that EGF can replace estradiol in supporting mammary gland development and the induction of estrogen-inducible genes (such as PR) in mice that lack ERα. By contrast, EGF had no significant effect on mammary gland development or PR induction in mice with intact ERα (WT mice and βERKO mice); this difference is likely to be the result of the near maximal stimulation of mammary gland development by ERα-mediated effects of E.

**Figure 4:**
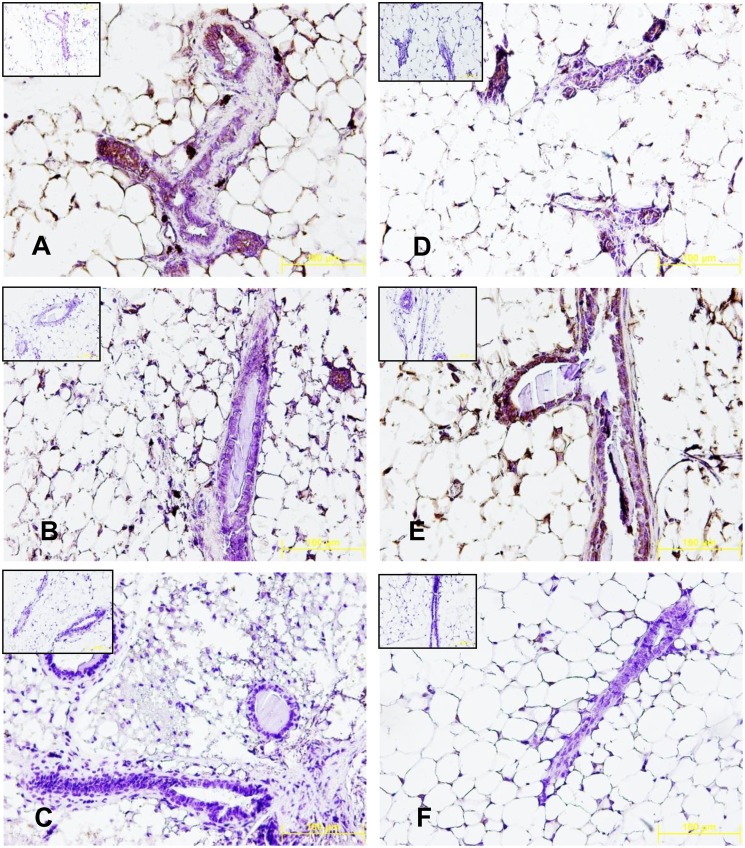
Effects of EGF on the induction of GPR30 by immunohistochemistry. C57 wild type, αERKO and βERKO mice were either injected with 25 ng EGF or vehicle subcutaneously daily for 5 days. The glands were dissected, fixed in formalin and 5 micron sections were processed for immunohistochemical staining. The sections were incubated with blocking reagent followed by GPR30 primary antibody and biotinylated secondary antibody. Antigen specific staining was visualized using DAB (brown) as a chromogen and nuclear counterstaining was done using hematoxylin (blue). Negative controls (sections not incubated with GPR30 antibodies) are shown as an inset for each condition. Results show that EGF induces GPR30 (brown) in the mammary glands of αERKO mice. A. Vehicle, Control; B. Vehicle, αERKO; C Vehicle, βERKO; D. EGF, Control; E. EGF, αERKO; F. EGF, βERKO.

GPR30 is involved in EGF transactivation, and has been shown to mediate cell proliferation and other responses to estrogen [23]. To determine if EGF can substitute for E in inducing the expression of GPR30, groups of WT mice, αERKO mice, and βERKO mice received daily subcutaneous injections of EGF (25 ng/dose) for 5 days. As shown in Figure 4, while mammary glands from WT showed some expression of GPR30, both αERKO and βERKO mice did not show any immunostaining (brown) for GPR30. Despite a basal difference in GPR expression between the glands derived from WT and βERKO mice, administration of EGF to either WT or βERKO mice did not have any effect on the enhancement of GPR30 expression. By contrast, administration of EGF to αERKO mice resulted in enhanced immunostaining suggesting a substantial upregulation of GPR30 protein expression in mammary ducts and alveoli. These results are consistent with our results for the effects of EGF on PR expression (Figure 3), and provide further evidence that, in the absence of functional ERα, EGF may substitute for estradiol mediated action in the mammary gland.

### Functional ERα is necessary for the development of mammary preneoplastic lesions

In view of the activity of EGF in supporting normal mammary growth and development, it was of interest to determine whether EGF can replace E in supporting the induction of mammary preneoplastic lesions (MAL) by the chemical carcinogen, DMBA. The gross appearance of MAL induced in organ culture by DMBA in WT mice pretreated with EGF + P was very similar to MAL induced by DMBA in WT mice pretreated with E + P. Furthermore, as seen in Table 1, the number of MAL induced in WT mice receiving E + P or EGF + P (13.3±7.8 lesions per gland in WT mice treated with E + P versus 12.8±6.8 lesions per gland in WT mice treated with EGF + P) was similar, once again suggesting that EGF can substitute for estrogen pretreatment in these mice.

**Table 1:** Effects of EGF on the induction of DMBA-induced MAL in mammary glands.

Genotype	Treatment	Number ofGlands	Glands withMAL (%)	MAL/Gland(Multiplicity ± SD)
WT	E + P	3	3 (100)	13.4+7.8
	EGF + P	4	4 (100)	12.8+6.8
αERKO	EGF + P	6	0	0
βERKO	E + P	7	5 (71)	4.7+3.9
	EGF + P	5	4 (80)	3.8+3.0

Although fewer mammary preneoplastic lesions were seen in βERKO mice than in WT mice, MAL were identified in DMBA-treated glands collected from βERKO mice that had been pretreated with EGF + P (Table 1). By contrast, no MAL were identified in mammary glands from αERKO mice pretreated with EGF + P. These results indicate that a functional ERα is necessary for MAL formation, and suggest that although EGF can support normal mammary ductal extension in the absence of E, EGF cannot support MAL formation.

### Differential Gene Expression in Mammary Glands from WT mice, αERKO mice, and βERKO mice

Patterns of gene expression in pooled RNA samples from cultured mammary glands from wild-type mice, αERKO mice, and βERKO mice pretreated with EGF + P were compared by microarray analysis. Of 28,665 genes that were expressed above background levels, 8,141 genes were differentially expressed in mammary glands from αERKO mice versus mammary glands from βERKO mice (Figure S1). In addition, 7,190 genes were differentially expressed in mammary glands from αERKO mice versus WT mice, and 4,218 genes were differentially expressed in mammary glands derived from βERKO mice versus WT mice.

Hierarchical clustering was performed for 11,588 genes that were differentially expressed in at least one comparison (Figure S2, Panel A) and 3927 genes that were identified as ERα responsive (Figure S2, Panel B). Principal Component Analysis (PCA) across samples was also performed using the 11,588 genes that were differentially expressed in at least one comparison (Figure S3).

Pathway analysis of the 11,588 differentially expressed genes demonstrated significant enrichment of 28 pathways (hypergeometric p values of <0.001). Of these, differential expression of 9 pathways was specific to αERKO mice. These included MAP kinase signaling pathway as well as signaling molecules affecting apoptosis. On the other hand, differential expression of 4 pathways was specific to βERKO mice, which included C-21 steroid metabolism (Table 2). The signaling molecules regulating cell proliferation were differentially regulated in these ERα and ERβ knockout conditions (data not shown).

**Table 2:** Analysis of EGF dependent αERKO and βERKO selective pathways in mouse mammary gland organ culture.

Differentially Expressed Pathway	p-Value
**αERKO Selective Pathways**	
MAPK signaling pathway	2.28E-05
Cytokine-cytokine receptor interaction	2.29E-05
Cell adhesion molecules (CAMs)	1.79E-04
Adherence junction	1.05E-03
Focal adhesion	2.21E-03
Regulation of actin cytoskeleton	3.50E-03
TGF-beta signaling pathway	3.98E-03
Tight junction	5.25E-03
Apoptosis	7.62E-03
**βERKO Selective Pathways**	
Taurine and hypotaurine metabolism	6.24E-03
C21-Steroid hormone metabolism	8.97E-03
Atrazine degradation	9.37E-03
Retinol metabolism	9.37E-03

## Discussion

Although ERα and ERβ functions have been extensively studied in various organs [24], [25], studies in ERα and ERβ knockout mice can provide additional insight into the roles of these ligand regulated transcription factors in both normal and neoplastic mammary development. For example, the primary mammary ducts, which are essential for the complete lobuloalveolar development of the mammary gland and for the initiation of lactation, are grossly underdeveloped in αERKO mice. By contrast, mammary development in βERKO mice is similar to that seen in wild-type mice, demonstrating the essential role of ERα (but not ERβ) in normal mammary gland growth and differentiation. Similar differential role of ERα and ERβ in mammary gland development has previously been reported [16], [17].

Pretreatment of αERKO mice with EGF + P supports the normal extension of mammary ducts throughout the fat pad when mammary glands are maintained in organ culture; this observation clearly demonstrates that EGF can substitute for estrogen-mediated ERα functions in normal mammary gland development and differentiation. However, although pretreatment with EGF can support normal ductal expansion in αERKO mice, it does not support the development of preneoplastic mammary lesions induced by DMBA. These data suggest that, despite clear evidence of interactions/cross-talk between E-dependent and EGF-dependent signaling pathways in normal mammary development, neoplastic development in the mouse mammary glands requires a functional ERα.

In addition to its effects in supporting normal mammary development and differentiation, EGF induced PR in the mammary glands of αERKO mice, supporting the results of previous studies in which findings where loss of ERα is often correlated with overexpression of EGFR [26], [22]. Moreover, it has been reported that GPR30, can mediate estrogen dependent signaling and EGFR transactivation by estradiol [27], This is consistent with our results showing that 5 day treatment of αERKO mice with EGF induced GPR30 in the mammary glands. However, present study suggests that there may be estrogen-specific molecular switch that may be required for transformation of mammary epithelial cells, which cannot be turned on by EGF by itself in the absence of estrogen-ERα interactions. Collectively these studies show that EGF can replace estradiol in the absence of ERα and may provide a basis for studies on the ERα and EGFR cross talk in normal mammal mammary gland development.

We analyzed microarray data generated from pooled RNA samples obtained from the mammary glands containing MAL derived from wild-type and βERKO mice and compared these with the RNA samples obtained from mammary glands (without MAL) from αERKO mice. PCA analysis showed major difference in the genomic profile between the RNA from MAL-negative glands of αERKO and MAL-positive glands of WT or βERKO mice. The PCA analysis also indicated that there is a close similarity between the glands containing MAL of βERKO and WT mice (Figure S3). This was further evident by selective differential expression by RNA isolated from the mammary glands of αERKO and βERKO mice as shown in Table 2.

In summary, the results described in this report provide direct evidence for the importance of ERα in normal mammary gland development and during neoplastic transformation. We showed that estrogen can be replaced by EGF for ER mediated function in the absence of ERα for the normal mammary gland development. However EGF in the absence of ERα was ineffective for the development of MAL. The results also indicated that like estrogen, in vivo treatment with EGF can also induce estrogen responsive gene, PR and EGF responsive GPR30 in the glands providing evidence for a cross talk between EGF and estrogen in normal mammary gland development. Finally the results from the microarray analyses indicated that the MAP Kinase signaling as well as estrogen dependent signaling resulting in reduced cyclin D and EGF dependent SRF, c-Jun and c-Fos expressions were responsible for the differences in cell proliferation between αERKO and WT or βERKO mice (data not shown). This is the first report to employ MMOC of the ERKO mice to delineate functional differences among αERKO, βERKO and wild type genotypes.

## Supporting Information

Figure S1 **Microarray analyses of RNA obtained from MAL containing glands from WT, αERKO and βERKO mice after MMOC.** Mammary glands were dissected from WT, αERKO or βERKO mice pretreated with 1 mg Progesterone and 25 ng EGF for 5 days. The glands were incubated with sequential combinations of hormones and carcinogen for 24 days as described in the Methods. This treatment schedule induces MAL in these glands. The glands were snap-frozen individually and RNA was extracted and Microarray analyses were performed on pooled RNA samples as described in the Methods. A. Venn diagram of genes >2-Fold in ERKO comparisons (Total 11,588 genes). The diagram indicates the number of genes that 723 genes are differentially expressed in all three comparisons. There are various distributions of number of genes that overlap between each combination.
(TIF)

Figure S2 **Differentially expressed genes in WT, αERKO and βERKO mice.** Genes (11588 genes) that are different in at least one comparison among the three genotypes (B) and 3927 genes >2-Fold in [αKO vs. WT] and [αKO vs. βKO] not [βKO vs. WT] (C). Genes are displayed as normalized to the median intensity of each array. Red = High expression, Yellow = Medium expression, Blue = Low expression. Results show that there is a close similarity between the expression of genes between WT and βERKO mice. However there are major differences between the αERKO mice and the two other genotypes. These results suggest that αER may be significantly more crucial for estradiol function as compared to ERβ (D).
(TIF)

Figure S3 **Principal Component Analysis.** Samples are displayed in respect to the two principal components present in 11,588 genes differentially expressed in at least one of three comparisons. Normalized expression differences between the αKO sample and the other two samples comprise the most differences in the data as PCA component 1. Normalized expression differences between WT and the two KO samples comprise the remainder of the differences in the data as PCA component 2.
(TIF)
