# Supplementary figures and images for: Differential Roles of ERα and ERβ in Normal and Neoplastic Development in the Mouse Mammary Gland
Source: PLoS One. 2014 Nov 18;9(11):e113175. doi: 10.1371/journal.pone.0113175 (PMC4236140; doi:10.1371/journal.pone.0113175)

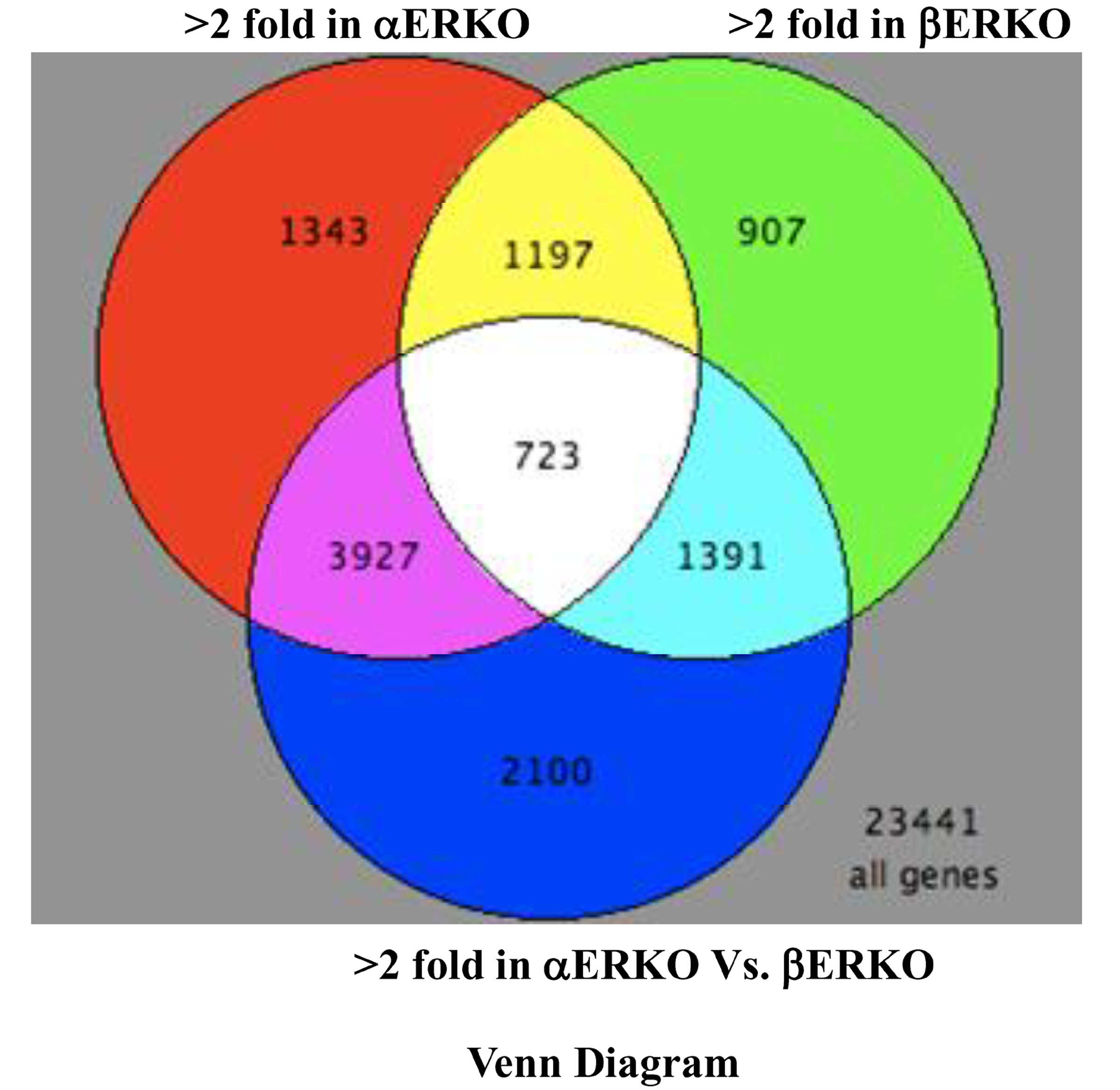

Supplement: Figure S1 — Microarray analyses of RNA obtained from MAL containing glands from WT, αERKO and βERKO mice after MMOC. Mammary glands were dissected from WT, αERKO or βERKO mice pretreated with 1 mg Progesterone and 25 ng EGF for 5 days. The glands were incubated with sequential combinations of hormones and carcinogen for 24 days as described in the Methods. This treatment schedule induces MAL in these glands. The glands were snap-frozen individually and RNA was extracted and Microarray analyses were performed on pooled RNA samples as described in the Methods. A. Venn diagram of genes >2-Fold in ERKO comparisons (Total 11,588 genes). The diagram indicates the number of genes that 723 genes are differentially expressed in all three comparisons. There are various distributions of number of genes that overlap between each combination. (TIF) [file pone.0113175.s001.tif]

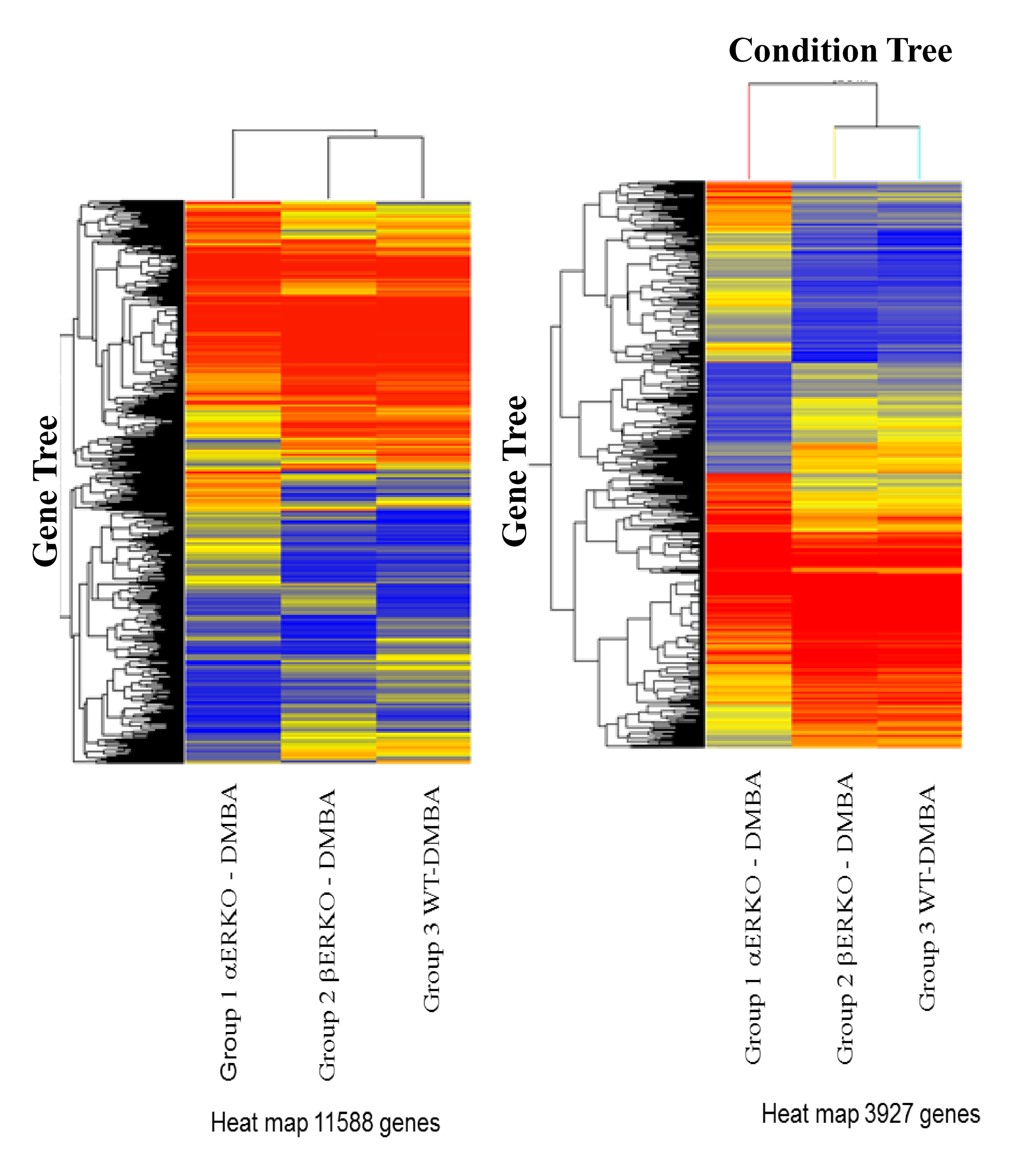

Supplement: Figure S2 — Differentially expressed genes in WT, αERKO and βERKO mice. Genes (11588 genes) that are different in at least one comparison among the three genotypes (B) and 3927 genes >2-Fold in [αKO vs. WT] and [αKO vs. βKO] not [βKO vs. WT] (C). Genes are displayed as normalized to the median intensity of each array. Red = High expression, Yellow = Medium expression, Blue = Low expression. Results show that there is a close similarity between the expression of genes between WT and βERKO mice. However there are major differences between the αERKO mice and the two other genotypes. These results suggest that αER may be significantly more crucial for estradiol function as compared to ERβ (D). (TIF) [file pone.0113175.s002.tif]

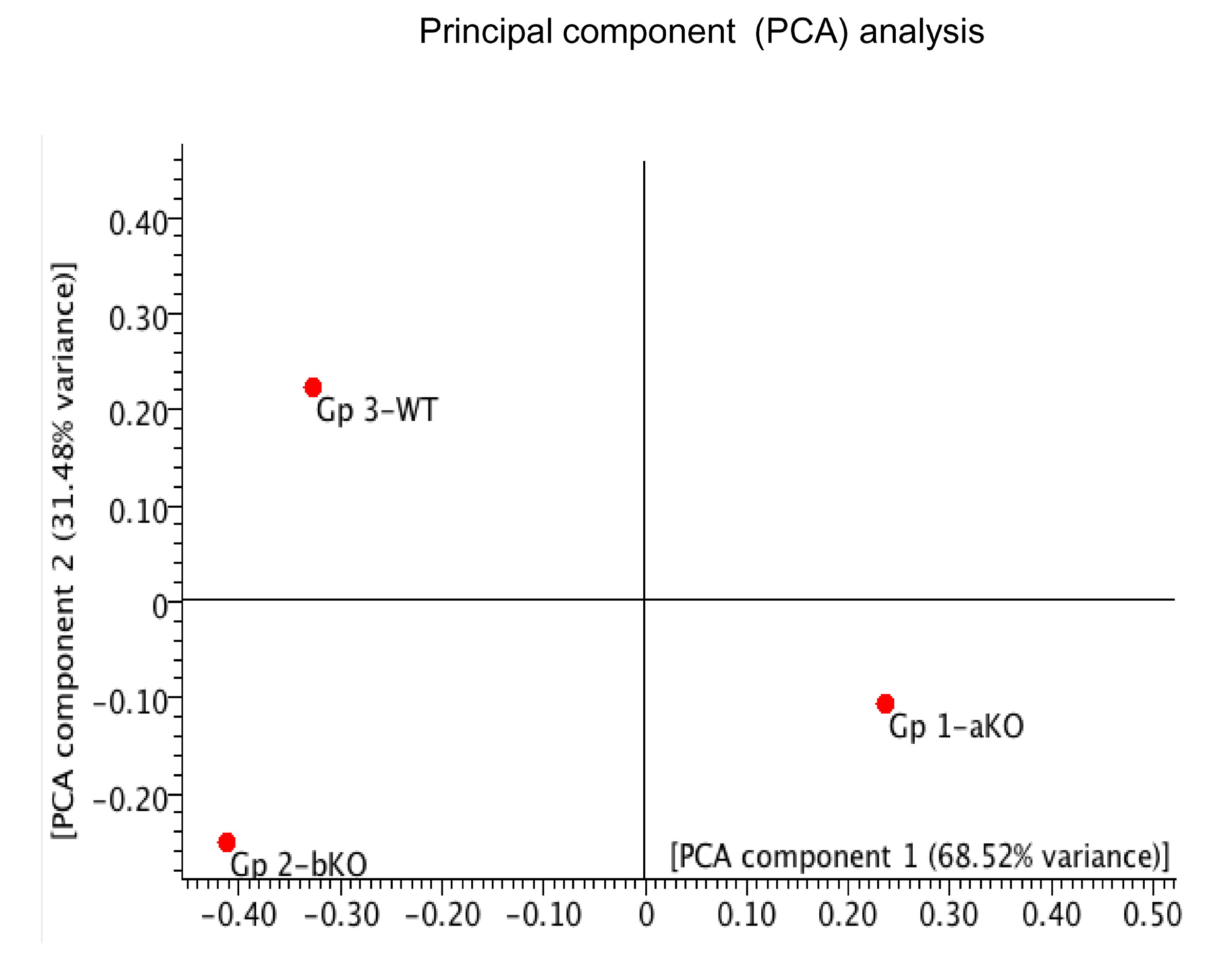

Supplement: Figure S3 — Principal Component Analysis. Samples are displayed in respect to the two principal components present in 11,588 genes differentially expressed in at least one of three comparisons. Normalized expression differences between the αKO sample and the other two samples comprise the most differences in the data as PCA component 1. Normalized expression differences between WT and the two KO samples comprise the remainder of the differences in the data as PCA component 2. (TIF) [file pone.0113175.s003.tif]
